# The contribution of residential greenness to mortality among men with prostate cancer: a registry-based cohort study of Black and White men

**DOI:** 10.1097/EE9.0000000000000087

**Published:** 2020-02-11

**Authors:** Hari S. Iyer, Linda Valeri, Peter James, Jarvis T. Chen, Jaime E. Hart, Francine Laden, Michelle D. Holmes, Timothy R. Rebbeck

**Affiliations:** a Department of Epidemiology, Harvard T. H. Chan School of Public Health, Boston, Massachusetts; b Department of Biostatistics, Columbia University Mailman School of Public Health, New York, New York; c Department of Population Medicine, Harvard Medical School and Harvard Pilgrim Health Care Institute, Boston, Massachusetts; d Department of Social and Behavioral Sciences, Harvard T. H. Chan School of Public Health, Boston, Massachusetts; e Department of Medicine, Channing Division of Network Medicine, Brigham and Women’s Hospital, Boston, Massachusetts; f Department of Environmental Health, Harvard T. H. Chan School of Public Health, Boston, Massachusetts; g Department of Medical Oncology, Dana Farber Cancer Institute, Boston, Massachusetts.

**Keywords:** Environmental epidemiology, Greenness, Mediation analysis, Prostate cancer, Racial disparities, Vegetation

## Abstract

Black men with prostate cancer (CaP) experience excess mortality compared with White men. Residential greenness, a health promoting contextual factor, could explain racial disparities in mortality among men with CaP.

**Methods:**

We identified Pennsylvania Cancer Registry cases diagnosed between January 2000 and December 2015. Totally, 128,568 participants were followed until death or 1 January 2018, whichever occurred first. Residential exposure at diagnosis was characterized using the Normalized Difference Vegetation Index (NDVI) with 250 m resolution. We estimated hazard ratios (HRs) using Cox models, adjusting for area-level socioeconomic status, geographic healthcare access, and segregation. To determine whether increasing residential greenness could reduce racial disparities, we compared standardized 10-year mortality Black-White risk differences under a hypothetical intervention fixing NDVI to the 75th percentile of NDVI experienced by White men.

**Results:**

We observed 29,978 deaths over 916,590 person-years. Comparing men in the highest to lowest NDVI quintile, all-cause (adjusted HR [aHR]: 0.88, 95% confidence interval [CI]: 0.84, 0.92, *P*_trend_ < 0.0001), prostate-specific (aHR: 0.88, 95% CI: 0.80, 0.99, *P*_trend_= 0.0021), and cardiovascular-specific (aHR: 0.82, 95% CI: 0.74, 0.90, *P*_trend_ < 0.0001) mortality were lower. Inverse associations between an interquartile range increase in NDVI and cardiovascular-specific mortality were observed in White (aHR: 0.90, 95% CI: 0.86, 0.93) but not Black men (aHR: 0.97, 95% CI: 0.89, 1.06; *P*_het_ = 0.067). Hypothetical interventions to increase NDVI led to nonsignificant reductions in all-cause (−5.3%) and prostate-specific (−23.2%), but not cardiovascular-specific mortality disparities (+50.5%).

**Discussion:**

Residential greenness was associated with lower mortality among men with CaP, but findings suggest that increasing residential greenness would have limited impact on racial disparities in mortality.

**What this study adds**

Racial disparities in prostate cancer are among the largest for any major cancer and could be mediated by neighborhood context. We conducted a cohort study in the Pennsylvania Cancer Registry to estimate associations between residential greenness and cause-specific mortality among Black and White men with prostate cancer. We observed statistically significant inverse associations between residential greenness and all-cause, prostate, and cardiovascular mortality. Though residential greenness does not appear to mediate disparities in all-cause and prostate cancer mortality, residential greenness could mediate cardiovascular mortality disparities due to differences in how Black and White men with prostate cancer interact with green spaces.

## Introduction

Cancer of the prostate (CaP) is the most commonly diagnosed cancer and the second leading cause of cancer-related death among men in the United States, accounting for one out of every 10 cancer deaths in men.^1^ In the US, Black men experience more than double the mortality from CaP compared with White men.^2^ Although racial gaps in access to CaP care have narrowed over time, disparities in mortality rates among men with CaP have persisted for as long as reliable registry data have been available.^3–5^ Causes of racial disparities in mortality among men with CaP are multifactorial, requiring a multilevel framework that considers genetic and lifestyle risk factors along with historic policies, environments, social attitudes, and community norms that differently shape experiences of Black and White men.^6–11^ Recent advances in epidemiologic methods have provided investigators with analytic tools to quantify the impact of social and environmental policy changes on racial disparities.^12–15^ Although most research to date on cancer disparities has focused on biological and social factors, few studies have investigated the mediating role of the built and contextual environment on racial disparities in cancer.^16^

A growing literature describes numerous health benefits of neighborhood greenness, defined as the extent of green, natural vegetation within a given area. More comprehensive than “green space,” the term neighborhood “greenness” includes all vegetation in a given area, regardless of type (e.g., parks, forests, gardens, and street trees). Neighborhood greenness is hypothesized to confer health benefits through promotion of healthy lifestyles and social cohesion, and reduction of harmful environmental exposures and biopsychosocial stressors.^17–20^ Cohort studies have reported inverse associations between neighborhood greenness and several diseases, including all-cause mortality, cardiovascular disease (CVD), and depression.^21–24^ In the US, neighborhoods with higher proportion of Black residents have lower levels of neighborhood greenness,^25^ suggesting that neighborhood greenness could mediate racial disparities in mortality among men with prostate cancer.

We studied the association between residential greenness and mortality in a cohort of Black and White men with CaP in Pennsylvania. Because earlier studies reported stronger associations between neighborhood greenness and specific causes of death,^21,22,26,27^ we assessed the magnitude of the association between residential greenness and all-cause mortality, prostate-specific mortality, and CVD mortality. We further evaluated whether the mortality disparity between Black and White men with CaP could be mediated by residential greenness.

## Methods

### Study design and participants

We used data from the population-based Pennsylvania Cancer Registry. We included 145,399 Black and White men with CaP diagnosed from 2000 to 2015 and followed them until death, 10 years postdiagnosis, or 1 January 2018, whichever came first. Participant addresses were geocoded using ArcGIS software version 10.2. We excluded cases who were diagnosed with in situ cancers (n = 69), missing address at diagnosis (n = 85), or missing stage or grade (n = 16,677). A total of 128,568 (88%) men with CaP were included in the study. The Institutional Review Board of Harvard T. H. Chan School of Public Health approved this study protocol. Because existing data sources were used, no written consent was required for participation in the study.

### Mortality assessment

CaP diagnoses were staged according to the 2000 Surveillance, Epidemiology, and End Results (SEER) summary staging guidelines.^28^ Race was extracted from facility medical records and included in data provided by state health providers to the Pennsylvania Cancer Registry. Each year, the Pennsylvania Cancer Registry conducts a Death Clearance in which reportable cause of death information from Pennsylvania Death Certificates is linked with Pennsylvania Cancer Registry files. If deaths occur out of state, linkage is done through data exchanges. Causes of death were categorized based on ICD-09 and ICD-10 codes. For CaP-specific mortality, we included deaths coded as 185 (ICD-09) and C61 (ICD-10). For CVD mortality, we included deaths coded as 390-459 and I00-I99.

### Exposure assessment

To estimate exposure to residential greenness for CaP cases at time of diagnosis, we used the normalized difference vegetation index (NDVI), a satellite-derived spatial measure of neighborhood greenness.^29^ NDVI values range from −1 to 1 and quantify the amount of infrared light absorbed vs reflected by plant life. NDVI values approaching 1 correspond to lush forests, close to 0 reflect barren areas, and below 1 indicate bodies of water. In this study, to focus specifically on associations related to green vegetation, we set values below 0 to missing. In sensitivity analysis, results were unchanged after applying this procedure. Moderate Resolution Imaging Spectroradiometer data capturing NDVI at a 250-m resolution were obtained using Google Earth Engine. We used Google Earth Engine’s cloud cover algorithm to extract the least cloudy image in January, April, July, and September for every year from 2000 to 2015, representing seasonal variation in residential greenness. Exposure was modeled using NDVI averaged over seasonal measures during calendar year of diagnosis (baseline), as well as cumulative updated average NDVI measurements over each participant’s entire follow-up period as a sensitivity analysis. Participants were assigned the baseline NDVI value for the 250 m^2^ pixel containing their residential address. Because we did not have information on participant mobility, in the case of cumulative updated average, we assigned the seasonal average NDVI over all years of follow-up at their residential address. We chose to model baseline NDVI as the primary exposure because we did not have time-varying information for any other variables in the analysis and wished to limit threat of collider stratification bias.^30,31^

We used Krieger’s ecosocial theory^6^ to develop a conceptual framework that integrates socioeconomic position, geographic barriers to access, along with demographic and clinical risk factors into our analytic framework. Area-level socioeconomic data at census Block Group level in 2000 were obtained from the National Historical Geographic Information System Database^32^ and spatially joined to cohort participant addresses using the R statistical package. We chose to link data at the census Block Group level because this is the smallest geographic unit for which the US census publishes data. In the 2010 census, Pennsylvania reported 9,740 Block Groups, which contained a median of 1,160 people (interquartile range [IQR]: 866–1,574). When Block Group data were not available (n = 101), we used census tract-level data.

### Statistical analysis

We estimated adjusted hazard ratios (aHRs) and 95% confidence intervals (CIs) for the association between NDVI and each of the mortality outcomes (all-cause, prostate, CVD) using multiple Cox proportional hazards models in SAS. NDVI was modeled using quintiles and as a continuous exposure using restricted cubic splines with three knots to test for nonlinearity. We chose to present results from models using quintiles alongside continuous NDVI because quintiles facilitate comparison of HRs between extremes of the NDVI distribution, mitigate the influence of outliers, and allow investigation of possible thresholds that could be used to guide a policy recommendation. When no evidence of nonlinearity was observed, we modeled continuous NDVI using a linear term scaled in units of IQR for the study population (0.14 U). We estimated *P* values for linear trend in categorical models using the median for each quintile. Because NDVI and CaP rates vary by urbanicity, we stratified our analyses by population density (≥1,000 people/mi^2^ vs. <1,000 people/mi^2^). This threshold was chosen to differentiate more rural settings from suburban and urban settings.

Because NDVI is tied to location and therefore socioeconomic status, we chose to control for possible confounding variables guided by the literature on socioeconomic position and health,^33^ racial disparities in prostate cancer,^5^ and neighborhood greenness and mortality.^34^ We considered advanced stage and marital status assessed at diagnosis as mediators because they could be interpreted as consequences of residential greenness exposure. We then sequentially adjusted for sets of confounding variables. In model 1, we stratified by age in 10-year categories and diagnosis year (categorical), and adjusted for race (Black vs. White); census Block Group median income (continuous: US$); median home value (continuous: US$); percent poverty (continuous); percent adults age 25 years and older with less than high school education (continuous); the joint race and income Index of Concentration at Extremes (ICE), a measure of inequality based on income- or race-based privilege in a given geographic area (quintiles)^35^; four indicator variables for receipt of care at a currently NCI-designated cancer center (The University of Pennsylvania, University of Pittsburgh Medical Center, Fox Chase Cancer Center, or Thomas Jefferson University Hospital); population density (continuous), distance between each participant’s geocoded address to the closest cancer center using road network distances (continuous, minutes, calculated using ArcMap 10.2). Although estimating road network distances makes strong assumptions (car-based transport, constant speed at road speed limit), this measure does allow rank ordering participants based on the proximity to cancer care. In model 2, we additionally adjusted for stage (categorical: localized, regional, distant) and grade (categorical: I–IV). In model 3, we further adjusted for marital status, using logistic regression models and Monte Carlo imputation with 10 repetitions to impute missing marital status (n = 46,519) conditional on the covariates used in model 1. We assessed whether primary associations varied by race (binary: Black vs. White), stage (binary: localized vs. regional/distant), and population density (≥1,000 people/mi^2^, <1,000 people/mi^2^). Though census Block Group socioeconomic variables were correlated (range [absolute value]: 0.43–0.84], no pair-wise correlation exhibited perfect collinearity (eTable 1; http://links.lww.com/EE/A71). Tests for effect modification were performed by fitting interaction terms between these modifiers and NDVI (continuous and as quintiles).

To evaluate the role of residential greenness as a potential mediator of racial disparities in cause-specific mortality among men with CaP, we estimated racial disparities among men with CaP after hypothetical interventions that fix NDVI for all participants to a specific value using previously described statistical methods.^13–15,36,37^ This approach assumes no unmeasured confounding of race and cause-specific mortality, no unmeasured confounding between residential greenness and cause-specific mortality, and correct model specification. Technical details are provided in eMethods 1; http://links.lww.com/EE/A71.

First, we fit the outcome model described above (Cox model 1) for each mortality outcome, omitting NDVI. Resulting model parameters were used to estimate 10-year mortality among Black and White men, standardized to covariates described above. The difference in standardized 10-year mortality for Black and White men with CaP was defined as the racial disparity. Because most Black men with CaP in our study lived in high population density areas, we repeated this procedure separately among men living in high and low population density areas. Next, we estimated the racial disparity that would remain after hypothetical interventions to fix NDVI to target values for all study participants. We again estimated expected racial disparities between Black and White men using our outcome model, with two additional parameters (continuous NDVI and an NDVI-race interaction). Bootstrapping with 500 repetitions was used to estimate 95% CIs.

Three levels of NDVI were chosen to set bounds on estimated changes in mortality among men with prostate cancer that could result from a policy change (1); the observed racial disparity with no change in NDVI, (2) the 25th percentile of NDVI among Black men with CaP (lower bound), and (3) the 75th percentile of NDVI among White men with CaP (upper bound). We then estimated the proportion of racial disparity that could be eliminated by implementing policy change (3).^12,38^ Details regarding sensitivity analyses for competing risks, and estimation of bounds for bias due to unmeasured confounding using E-values^39^ are provided in eMethods 1; http://links.lww.com/EE/A71.

## Results

**Figure 1.**
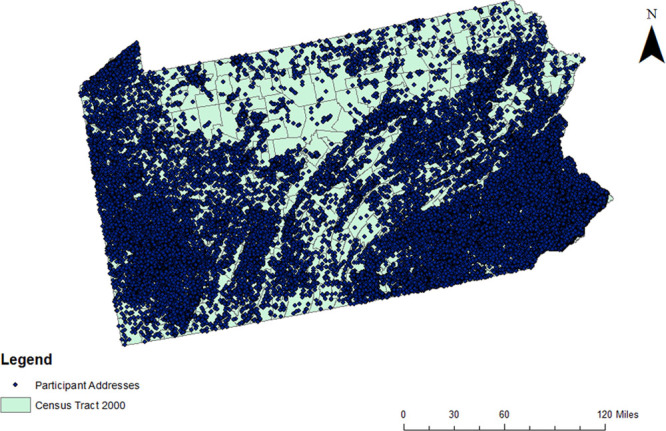
Participant residential address locations in Pennsylvania Cancer Registry prostate cancer cohort study from 2000 to 2015.

**Figure 2.**
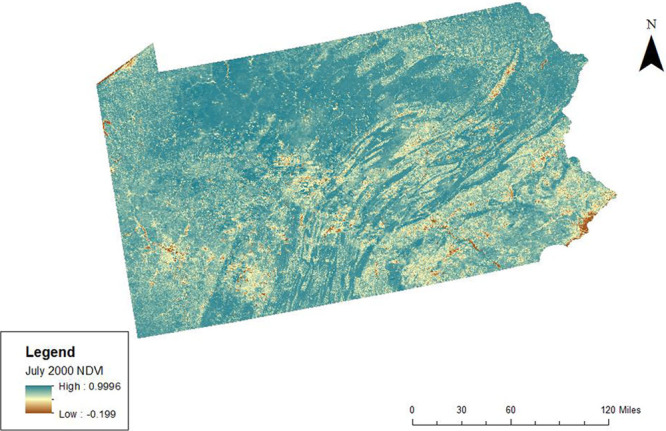
Normalized difference vegetation index July 2000 values at Pennsylvania Cancer Registry participants’ residential address locations from 2000 to 2015.

After exclusions, we observed 29,978 deaths over 916,590 person-years of postdiagnosis follow-up. Study population characteristics are presented in Table 1 overall and by NDVI in the year before diagnosis. Median age at diagnosis was 66 and did not vary by quintile of NDVI. Black men made up 11% of the study population and were less likely than Whites to reside in neighborhoods in the highest quintile of NDVI (NDVI Q1: 33% vs. Q5: 3%). Most participants were diagnosed with localized disease (85%). Participants in greener neighborhoods (Q5) had lower population density, higher census Block Group income and median home value than participants in less green neighborhoods (Q1). Study participants were concentrated in the Southeast and Western parts of Pennsylvania, corresponding to the Pittsburgh and Philadelphia metropolitan areas where NDVI was relatively lower than in other regions of the state (Figs. 1 and 2). CVD was leading cause of death (n = 7,677), followed by CaP (n = 6,515).

**Table 1.**
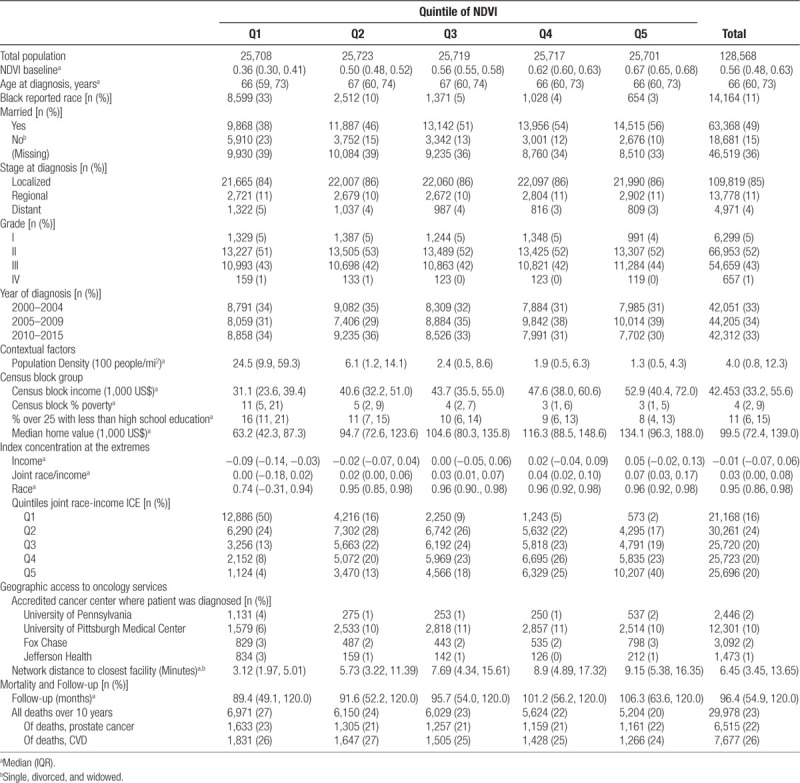
Descriptive characteristics of Pennsylvania Cancer Registry Cohort stratified by quintile of baseline NDVI quintile, from 2000 to 2015.

In adjusted analysis, we observed statistically significant inverse associations between NDVI in the year of diagnosis and rates of mortality using quintiles and continuous exposure parameterizations (Table 2). Tests for splines were not significant, so we assumed linear dose response between continuous NDVI and mortality. When considering confounding factors (model 1), there was a 12% lower rate of all-cause mortality comparing participants with NDVI Quintile 5 to 1 (Q5 to 1) (aHR: 0.88, 95% CI: 0.84, 0.92, *P*_trend_ < 0.0001). This association was similar for prostate-specific mortality (aHR: 0.88, 95% CI: 0.80, 0.98, *P*_trend_ = 0.0021), but the relative stability of HR estimates across NDVI quintiles suggests that this result should be interpreted with caution. For CVD mortality, there was an 18% lower rate comparing NDVI Q5 to 1 (aHR: 0.82, 95% CI: 0.74, 0.90, *P*_trend_ < 0.0001).

**Table 2.**
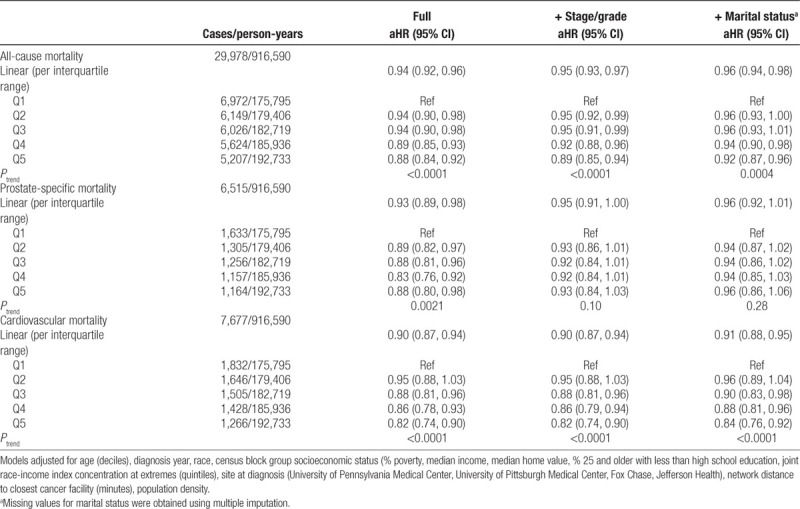
Cox proportional hazards models for association between NDVI at diagnosis and cause-specific mortality among Pennsylvania prostate cancer patients diagnosed between 2000 and 2015.

The associations with prostate-specific mortality were attenuated in models additionally adjusting for stage and grade (aHR NDVI Q5 to 1: 0.93, 95% CI: 0.84, 1.03) and then marital status (aHR NDVI Q5 to 1: 0.96, 95% CI: 0.86, 1.06). Adjusting for stage and grade did not result in major changes in inference with respect to all-cause mortality or CVD mortality. However, adjusting for marital status resulted in modest attenuation of the association with all-cause mortality (aHR NDVI Q5 to 1: 0.92, 95% CI: 0.87, 0.96), but not CVD mortality.

In stratified analyses, we found no evidence of effect modification by race, stage, or population density with respect to all-cause mortality (Table 3). The inverse association between an IQR increase in continuous NDVI and prostate-specific mortality was stronger among participants with localized (aHR: 0.92, 95% CI: 0.87, 0.97) compared with distant CaP (aHR: 0.98, 95% CI: 0.93, 1.03, *P*_het_ = 0.032). In addition, the inverse association was stronger among participants in high (aHR: 0.88, 95% CI: 0.83, 0.93) compared with low (aHR: 0.96, 95% CI: 0.91, 1.01) population density areas (*P*_het_ = 0.028). There was no association between continuous NDVI and CVD mortality among Black men with CaP (aHR: 0.97, 95% CI: 0.89, 1.06), but there was an inverse association among White men with CaP (aHR: 0.90, 95% CI: 0.86, 0.93, *P*_het_ = 0.067), suggesting increasing levels of NDVI could increase disparities by preferentially benefiting White but not Black men with CaP.

**Table 3.**
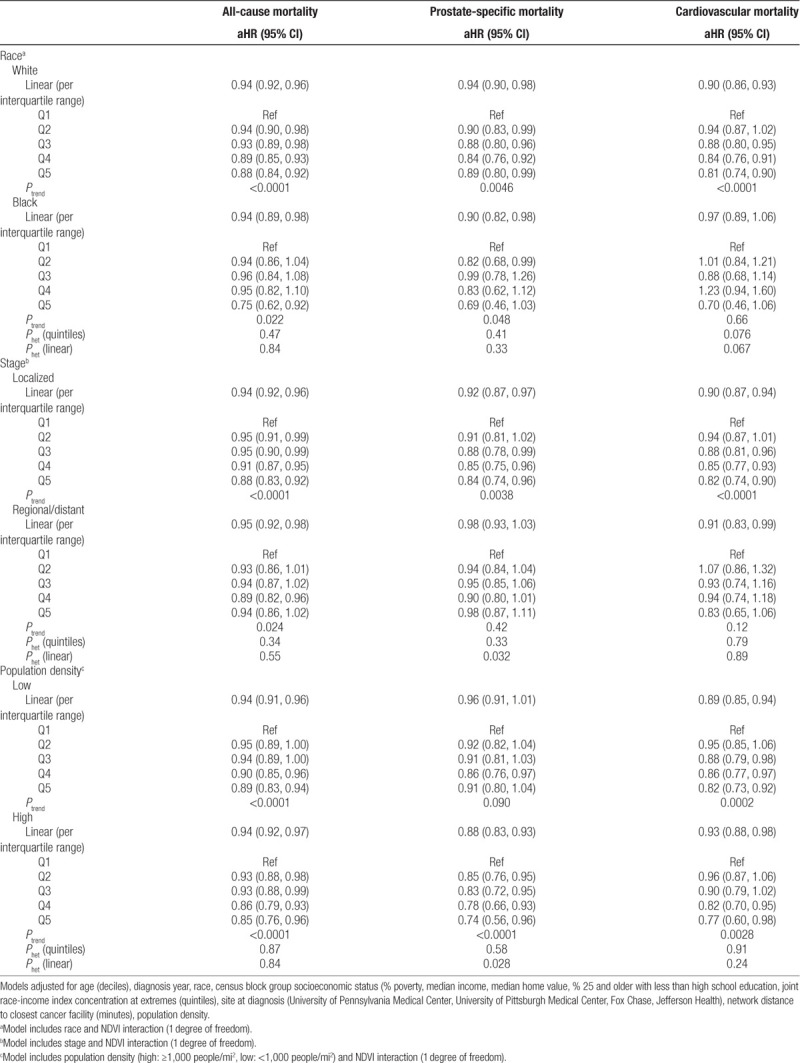
Cox proportional hazards models for association between NDVI at diagnosis and cause-specific mortality among Pennsylvania prostate cancer patients diagnosed between 2000 and 2015, stratified by race, stage and population density

**Figure 3.**
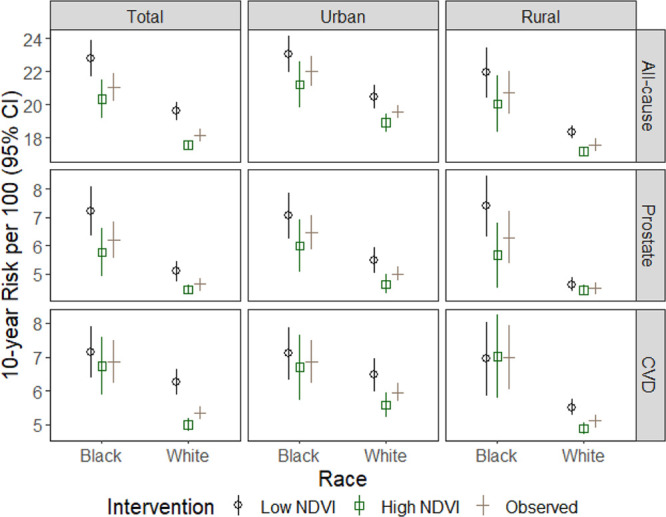
Standardized 10-year cause-specific (all-cause, prostate-, and cardiovascular-specific) mortality risk and 95% CIs under three scenarios^a^ of NDVI at diagnosis among Black and White men with prostate cancer in Pennsylvania. ^a^Scenario 1: observed NDVI, Scenario 2: low NDVI (25th percentile of Black men with CaP), Scenario 3: high NDVI (75th percentile of White men with CaP), CVD, cardiovascular-specific mortality, (Urban: ≥1,000 people/mi^2^, Rural: <1000 people/mi^2^).

Racial disparities in 10-year mortality without adjustment for NDVI were greatest for all-cause mortality, resulting in 29.3 excess deaths per 1,000 (95% CI: 22.1, 36.5) among Black men with CaP, and least for CVD mortality (11.5, 95% CI: 6.4, 16.7 excess deaths per 1,000). Disparities were greater in low (all-cause: 33.9, 95% CI: 20.9, 47.8; prostate: 22.1, 95% CI: 13.0, 31.2; CVD: 16.8, 95% CI: 7.3, 26.3 per 1,000) compared with high population density areas (all-cause: 25.1, 95% CI: 15.2, 35.0; prostate: 15.1, 95% CI: 8.1, 22.1; CVD: 8.5, 95% CI: 1.6, 15.3 per 1,000). There were no statistically significant differences in racial disparities among men with CaP after hypothetical interventions fixing residential NDVI to the 25th percentile (Black), observed values of NDVI, or the 75th percentile among (White) (Table 4). Fixing NDVI to the 75th percentile (White) resulted in the lowest cause-specific mortality, and fixing NDVI to the 25th percentile (Black) resulted in the highest cause-specific mortality in all scenarios except for CVD mortality among Black men in low population density areas (Fig. 3).

**Table 4.**
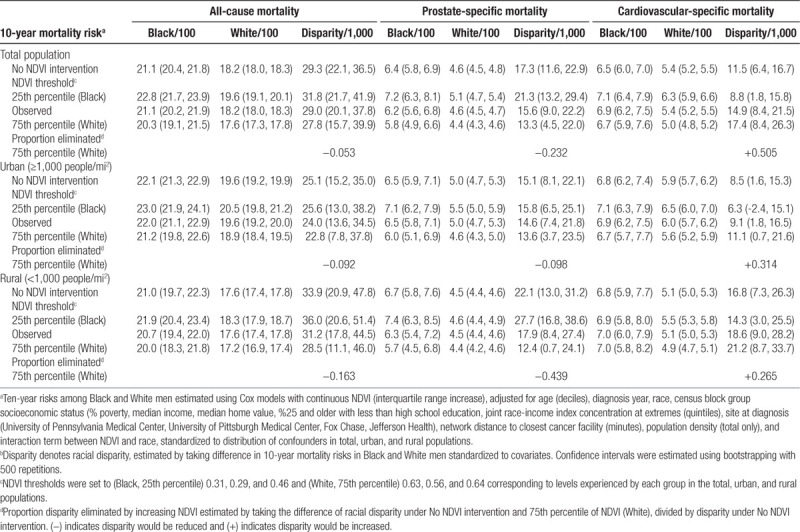
Cause-specific 10-year mortality risks^a^, disparities^b^, and 95% CIs under three levels of NDVI at diagnosis among Black and White men with prostate cancer in Pennsylvania, 2000 to 2015.

Estimated proportions of racial disparity in mortality that would be eliminated by fixing residential greenness to the 75th percentile of NDVI (White) were modest for all-cause (5.3%) and prostate-specific (23.2%) mortality. However, for CVD mortality, we estimated a relative 50.5% increase in the racial disparity after this hypothetical intervention (Table 4). These findings are consistent with results from our race-stratified models, in which NDVI was associated with lower CVD mortality among White but not Black men with CaP. Stratification by population density preserved these patterns, though estimated proportions of racial disparities eliminated for all-cause and prostate mortality were greater in low compared with high population density areas (Table 4).

In sensitivity analysis for competing risks, results for stratified associations between NDVI and prostate- and CVD-specific mortality resulted in slightly weaker estimates compared with primary results and no change to inference so we did not use competing risk models for our main analysis (eTable 2; http://links.lww.com/EE/A71). E-values summarizing bounds of bias due to unmeasured confounding for our primary effect estimates are provided in eTable 3; http://links.lww.com/EE/A71. Effect sizes for the association between socioeconomic status, a likely confounding variable, and mortality among men with CaP from previous registry-based studies range from 1.14 to 1.52.^40^ E-values for all-cause mortality and prostate-specific mortality lie within this range, meaning that if an unmeasured factor exhibited patterns of association with NDVI and mortality similar to that of socioeconomic status, adjusting for that factor could explain away these results. However, this unmeasured factor would need to be sufficiently correlated with NDVI and mortality even after adjusting for the demographic, socioeconomic, and geographic access variables already included in our analysis. Our strongest e-values corresponding to the HR for CVD-mortality comparing men in Q5 to Q1 are 1.75 for point estimate, and 1.47 for CI, suggesting that these results are unlikely to be explained by unmeasured confounding bias. Associations between cumulative updated average NDVI and mortality exhibited non-linear dose response, with increased all-cause mortality and prostate-specific mortality in the lowest and highest quintiles of NDVI (eTable 4; http://links.lww.com/EE/A71).

## Discussion

In this cohort of Black and White men with CaP, we observed inverse associations between NDVI and lower all-cause, prostate-, and CVD-specific mortality after adjusting for demographics, neighborhood socioeconomic context, and geographic healthcare access. Our results suggest that increasing levels of residential greenness could result in modest, nonsignificant decreases in racial disparities in all-cause and prostate-specific mortality. However, we estimated increases in racial disparities in CVD mortality among men with CaP after hypothetical interventions to increase residential greenness. In our sensitivity analysis using cumulative updated average NDVI, we observed different dose-response patterns compared with analyses using NDVI at time of diagnosis. Increased all-cause and prostate specific mortality observed with increasing cumulative updated average NDVI could be attributable to reverse causation, resulting from tree planting and greening interventions such as Philadelphia’s “Green Works” program, implemented from 2009 to present. These interventions were targeted precisely at those urban areas which were most deprived and experienced worse outcomes during the study period.^41^

Although few studies have reported associations between neighborhood greenness and mortality among men with CaP, our findings are consistent with results from earlier prospective population-based and occupational cohort studies in the US, Canada, and Europe, which have also reported inverse associations between neighborhood greenness and all-cause mortality.^21,23,26,27^ Most men in our study were diagnosed with localized CaP. The 10-year survival is relatively high among these men, and deaths from prostate cancer are few relative to deaths from other causes like CVD.^42,43^ This suggests mortality risks for these men could be similar to the general population. Cohort studies in Canada, Europe, and the US have also reported inverse associations between neighborhood greenness and cardiovascular mortality.^22,23,26,27,44^ Though we did not have data to evaluate lifestyle risk factors, prior research shows that physical activity is associated with lower mortality risk,^45–47^ and obesity is associated with higher risk^48,49^ among men with CaP. In our study, CVD-specific mortality was the leading cause of death among men with CaP, so inverse associations between residential greenness and mortality reported here could be due to reduced CVD-specific mortality, possibly through pathways related to physical activity and obesity.^17,18,20^ Empirical estimates of the proportion of inverse association between NDVI and mortality mediated by physical activity are few, with a single large prospective cohort of female nurses reporting 2.1% mediated by physical activity (based on questionnaire responses) using a 1,250 m buffer for NDVI.^21^ Future investigations using more precise estimates of physical activity, for example, through accelerometry, may yield stronger relationships.^50^

The second question we sought to answer was whether increasing residential greenness could reduce racial disparities in mortality among men with CaP. No differences in the association between NDVI and either all-cause or prostate-specific mortality comparing Black to White men with CaP were observed. However, for CVD mortality, we observed an inverse association with NDVI in White but not Black men. Wide confidence intervals for the cause-specific racial disparities from our simulation-based approach limited our ability to statistically evaluate differences in disparities under hypothetical interventions to fix NDVI to different thresholds. However, estimates of the proportion of disparity eliminated suggest that increasing residential greenness could lead to modest reductions in disparities in all-cause mortality. Estimated reductions in racial disparities for CaP mortality were offset by increases in disparities for CVD mortality. Cohort studies in the general population from Canada and Europe looking at all-cause and CVD mortality have also reported stronger inverse associations among high income or privileged racial groups.^26,27^ Better understanding of how contextual environment and CaP outcomes vary by race in different US and global settings will be essential to informing policy interventions.

Although we lacked data to explain racial differences in the association between NDVI and CVD mortality, the literature on differing patterns of park use between Black and White men and women offers some guidance. Parks are a major contributor to urban neighborhood greenness. The ways in which Black and White men experience neighborhood greenness could be different, which in turn could have consequences for potential health benefits of exposure to high levels of greenness. Parks in predominantly Black neighborhoods may be used less frequently due to fewer resources for security and maintenance.^51^ Black men in the US may use parks differently because they were historically excluded from public parks through segregation.^52,53^ Residents’ perception of higher crime rates, lower levels of walkability, and lack of upkeep could make parks less welcoming for physical activity and socializing, particularly for older community members.^54–56^ Surveys of park users in the US have found that Black community members often cite greater obstacles to using parks compared with White users, including feeling unwelcome, inconvenient schedules, and financial barriers.^53,57^ These findings suggest that merely introducing neighborhood green spaces in these communities, without any attempts to ensure that the space fits the needs and social mores of that community, could fail to produce any health benefits.

Results from our study should be interpreted in light of its limitations. This study was conducted in the state of Pennsylvania, which has a unique history, geography, and racial composition. Thus, our results may not be generalizable to dissimilar populations. NDVI is a popular objective measure of neighborhood and residential greenness, but does not capture quality or accessibility of green spaces, which may be necessary to inform appropriate interventions. It is possible that by focusing on residential greenness at diagnosis, we fail to capture other possibly meaningful sources of greenness exposure that occur in work or recreational settings, or at different residences during follow-up, leading to measurement error with respect to total greenness exposure. We did not have measurements of screening, health insurance, diet, and lifestyle factors (including physical activity) at diagnosis, which could influence neighborhood selection and mortality, leading to unmeasured confounding (a threat to all observational studies). However, assuming that socioeconomic status lies upstream of these proximal confounding variables, controlling for socioeconomic status should mitigate this bias. Our sensitivity analysis using E-values suggests that unmeasured variables would be unlikely to completely explain our effect estimates, particularly for CVD mortality. If lifestyle factors lie on the causal path between neighborhood greenness and mortality, we would not adjust even if those data were available. The same argument applies to treatment and quality of life measures postdiagnosis-although they may be important mediators, we lacked data to evaluate these pathways. Measurements of residential air pollution, which has been proposed as a confounder of the greenness-mortality association, were unavailable. However, other large cohort studies reveal that further adjusting for air pollution after adjustment for demographic and socioeconomic variables does not lead to major changes in the estimate of inverse association between NDVI and mortality.^22,26^ Strengths of our study include a cohort design with long follow-up, a large, racially diverse population, adjustment for major sociodemographic, clinical, and contextual environmental confounders, and analysis of the contribution of environment to racial disparities.

In conclusion, we report an inverse association between residential greenness and rate of all-cause, prostate- and CVD-specific mortality among men with CaP in Pennsylvania. Although interventions to set thresholds of residential greenness could have limited impact on reducing racial disparities, increases in greenness were associated with reduced all-cause and prostate-specific mortality rates among both Black and White men with CaP. Enhanced understanding of differences in how Black and White men interact with green spaces could inform targeted nature-based interventions to allow all men with CaP to experience those benefits.

## Conflict of interest statement

M.D.H. declares receipt of aspirin for trial NCT 02927249 from Bayer AG, consulting for Arla Foods, United States Social Security Administration, VISIONS Inc., and service on Cambridge Savings Bank’s advisory board. The other authors have no conflicts to report.

## Acknowledgments

We are grateful to the men in Pennsylvania who contributed data to the Pennsylvania Cancer Registry for this analysis. We also thank our colleagues at the Pennsylvania Cancer Registry and the Brigham and Women’s Hospital for administrative support. We thank Jeffrey C. Blossom at the Harvard Center for Geographic Analysis for providing technical support with Geographic Information Systems for this analysis.
